# Cumulative non-high-density lipoprotein cholesterol burden and risk of atherosclerotic cardiovascular disease: a prospective community-based study

**DOI:** 10.3389/fcvm.2023.1105342

**Published:** 2023-05-18

**Authors:** Xu-Min Guan, Hong-Po Shi, Shuang Xu, Yue Chen, Rong-Feng Zhang, Ying-Xue Dong, Lian-Jun Gao, Shou-Ling Wu, Yun-Long Xia

**Affiliations:** ^1^ Department of Cardiology, First Affiliated Hospital of Dalian Medical University, Dalian, China; ^2^ Department of Cardiology, Beijing Jingmei Group General Hospital, Beijing, China; ^3^ Department of Cardiology, Kailuan General Hospital, Tangshan, China

**Keywords:** non-HDL cholesterol, cumulative exposure, burden, atherosclerotic cardiovascular disease, risk factor

## Abstract

**Background:**

The relationship between cumulative non-high-density lipoprotein cholesterol (non-HDL-C) burden and atherosclerotic cardiovascular disease (ASCVD) remains unclear

**Objective:**

To prospectively examine the association between cumulative non-HDL-C burden and ASCVD risk in the Kailuan cohort of China.

**Methods:**

A total of 49,679 subjects who were free of ASCVD participated in three consecutive examinations in 2006, 2008 and 2010 were enrolled. Duration and concentration of cumulative exposure to non-HDL-C (cumNon-HDL-C) were respectively used to estimate the extent of cumulative non-HDL-C burden. The participants were divided into four groups according to durations of cumNon-HDL-C (0, 2, 4 and 6 years) and five groups according to the quintiles of cumNon-HDL-C concentration (<10.93, 10.93–12.68, 12.69–14.32, 14.33–16.72 and ≥16.73 mmol/L). Cox regression models were used to analyze the influence of cumulative non-HDL-C burden on ASCVD risk.

**Results:**

We identified 1,134 incident ASCVD cases during a mean of 4.89 years of follow-up. Multivariable adjusted analysis revealed that compared with no exposure, cumNon-HDL-C duration 2, 4 and 6 years increased ASCVD risk by 26% (HR: 1.26, 95% CI: 1.07–1.47), 56% (HR: 1.56, 95% CI: 1.31–1.86) and 91% (HR: 1.91, 95% CI: 1.59–2.31) respectively; The hazard ratios (HRs) for the fourth and fifth versus lowest quintile of cumNon-HDL-C concentration were 1.25 and 1.72 for ASCVD. Each standard deviation increment in cumNon-HDL-C concentration was associated with a 10% increased risk of ASCVD.

**Conclusion:**

Long-term and higher cumNon-HDL-C were all significantly associated with an increased risk of ASCVD independent of single non-HDL-C level.

## Introduction

Non-high-density lipoprotein cholesterol (non-HDL-C) can be calculated as total cholesterol minus high-density lipoprotein cholesterol, which is an estimate of the total amount of proatherogenic apolipoprotein B (ApoB)-containing lipoproteins (1, 2). Such proteins include triglyceride-rich particles in very low-density lipoproteins and their remnants, intermediate-density lipoproteins, lipoprotein(a), and low-density lipoproteins (1, 2). Numerous studies have shown that non-HDL-C is significantly associated with risk of atherosclerotic cardiovascular disease (ASCVD) (3–7). Non-HDL-C was also demonstrated to be a better risk indicator for cardiovascular events than low-density lipoprotein cholesterol (LDL-C) (4, 8–10) and is therefore recommended by current US and European guidelines for cardiovascular risk estimation (11, 12). However, most studies have only focused on the prognostic value of the baseline non-HDL-C measured at only a single point time. Actually, non-HDL-C may be easily affected by many factors, such as age change, diet, healthy lifestyle habits, the use of lipid-lowering drugs and some diseases. A single measurement of non-HDL-C may be not sufficient to convincingly demonstrate the effect of long-term non-HDL-C on ASCVD.

Cumulative exposure can accurately reflect the long-term effect of a factor on an individual (13–15). It is unclear whether cumulative non-HDL-C burden such as duration and concentration of cumulative exposure to non-HDL-C (cumNon-HDL-C) provides clinically significant prognostic information regarding ASCVD risk. Therefore, we explored the association between cumulative non-HDL-C burden and ASCVD incidence in a Chinese population from Kailuan cohort.

## Methods

### Selection of study population

**Figure 1:**
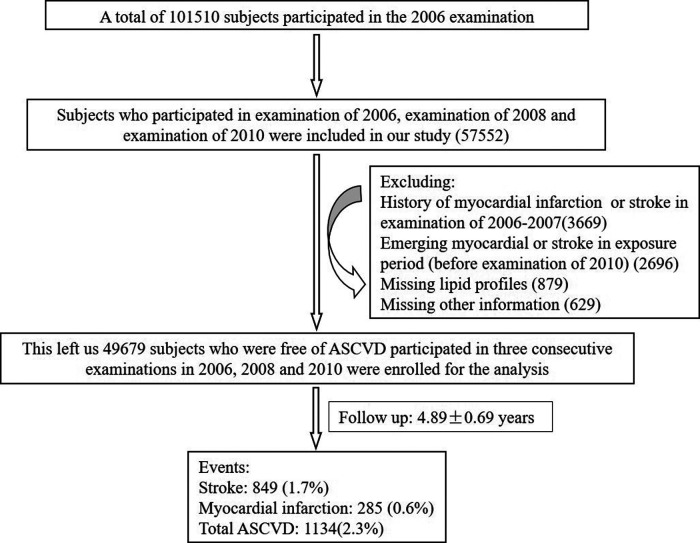
The flowchart of the study.

The data of this prospective study were derived from the Kailuan Study, which was a prospective, community-based study in Tangshan, an industrial city in China. A total of 101,510 participants participated in the first survey including a standardized questionnaire, physical examination and laboratory tests in 2006. We performed re-examinations biennially in 2008, 2010, 2012, and 2014. Participants we enrolled should completed the first three surveys (examination of 2006, examination of 2008 and examination of 2010) to calculate the cumulative burden of non-HDL-C. There were 57,552 subjects initially included in our study. We excluded 3,669 participants due to a history of ASCVD such as myocardial infarction or stroke at the baseline; 2,696 participants due to emerging myocardial or stroke in exposure period (2006–2010); 879 participants due to missing lipid profiles and 629 participants due to missing other information. This left a total of 49,679 participants without ASCVD at baseline for the final analysis (Figure 1). This study was performed according to the guidelines of the Helsinki Declaration and was approved by the Ethics Committee of Kailuan General hospital. All participants signed informed consents.

### Data collection and assessment of variables

Information on age, gender, education, income, marital status, smoking status, alcohol intake, physical activity, medical history included hypertension, diabetes mellitus, cardiovascular disease and active treatment such as hypoglycemic, antihypertensive and lipid-lowing agents was obtained via standardized questionnaires. Blood pressure (BP) was measured twice from the seated position at rest and the average of two readings was used for analysis. Hypertension was defined as systolic BP ≥140 mmHg, diastolic pressure ≥90 mmHg (16) or use of antihypertensive agents in the last 2 weeks regardless of BP status. Diabetes mellitus was defined as fasting plasma glucose (FPG) level ≥7.0 mmol/L, or 2-h plasma glucose (2-h PG) ≥11.1 mmol/L (17) or use of insulin or oral hypoglycemic agents. Body weight and height were measured by trained nurses during surveys. Body mass index (BMI) was calculated as body weight in kilograms divided by the square of height in meters.

Blood samples were collected from participants in the morning of the survey after an overnight fast and stored in vacutainer tubes containing ethylene diamine tetraacetic acid. Lipid profiles included LDL-C, high-density lipoprotein cholesterol, total cholesterol and triglycerides were measured enzymatically (Mind Bioengineering Co. Ltd, Shanghai, China). Non-HDL-C levels were determined by subtracting serum high-density lipoprotein cholesterol levels from total cholesterol. FBG was measured with the hexokinase/glucose-6-phosphate dehydrogenase method.

### Definition of cumulative non-HDL-C burden

To precisely determine cumulative non-HDL-C burden on ASCVD incident, duration and concentration of cumNon-HDL-C were used to represent the extent of non-HDL-C exposure of an individual. We applied 4.1 mmol/L as cut-off value of non-HDL-C exposure. Duration of cumNon-HDL-C was defined as 0, 2, 4 and 6 years according to non-HDL-C no exposure, exposure in one survey, two surveys and all surveys during three physical examinations. CumNon-HDL-C concentration was calculated as the summed average non-HDL-C levels for each pair of consecutive examinations multiplied by the years between the consecutive visits: [(non-HDL-C_1_ + non-HDL-C_2_)/2 × time_1–2_] + [(non-HDL-C_2_ + non-HDL-C_3_)/2 × time_2–3_] where non-HDL-C_1_, non-HDL-C_2_ and non-HDL-C_3_ indicate non-HDL-C level at the first, second and third examination and time_1–2_, time_2–3_ indicate the time interval between the two adjacent examinations respectively. The participants were divided into four groups according to durations of cumNon-HDL-C (0, 2, 4 and 6 years) and five groups according to the quintiles of cumNon-HDL-C concentration (<10.93, 10.93–12.68, 12.69–14.32, 14.33–16.72 and ≥16.73 mmol/L).

### Follow-up and ASCVD ascertainment

We followed up each participant from the third medical examination in 2010 until the occurrence of ASCVD including myocardial infarction or stroke, death or December 31, 2014. Ascertainment of incident ASCVD was defined according to ICD-10 system. All participants were linked to the Municipal Social Insurance Institution database and Hospital Discharge Register to identify potential cases of incident ASCVD. Information on death was obtained from the State Vital Statistics Office.

### Statistical analysis

Statistical analyses were performed using SAS version 9.4 (SAS Institute, Inc, Cary, North Carolina, USA) and SPSS 13.0 (SPSS Inc, Chicago, IL, USA). *P*-values were based on a two-sided test of significance and *P* < 0.05 was considered statistically significant. Continuous variables were described by mean ± standard deviation (SD) and compared using one-way ANOVA analysis. Categorical variables were described by percentages and compared via Chi-Squared tests. Cox regression model was used to estimate hazard ratios (HR) and 95% confidence intervals (CI) for ASCVD incidence based on cumulative burden of non-HDL-C by adjusting for the following confounders including age, gender, cigarette smoking, alcohol consumption, physical activity, systolic BP, FBP, BMI and lipid-lowing drugs use at baseline. For sensitivity analysis, the participants who used antihypertensive drugs, hypoglycemic drugs and lipid-lowing drugs were deleted separately or simultaneously in the above model.

## Results

### Baseline characteristics of the participants

The baseline information was the data from the third medical examination in 2010. This study included 49,679 participants (Figure 1). The mean age was 52.44 ± 11.75 years, 37,851 participants (76.2%) were men. The demographic and clinical characteristics of these participants with different cumNon-HDL-C durations and cumNon-HDL-C concentrations are summarized in Tables 1, 2 respectively. In general, participants with longer cumNon-HDL-C duration (Table 1) or higher cumNon-HDL-C concentration (Table 2) were older, more likely to have hypertension and diabetes mellitus, with a higher non-HDL-C, total cholesterol and triglyceride concentrations, with a higher BMI, more likely to use lipid-lowing drugs, but to engage in more physical activity, which may be related to medical guidance.

**Table 1:** Characteristics of participants by cumNon-HDL-C duration.

Characteristics	Total	Durations of cumNon-HDL-C			
		0 year	2 years	4 years	6 years
Subjects, *n*%	49,679	30,520 (61.4)	10,026 (20.2)	5,586 (11.2)	3,547 (7.1)
ASCVD, *n*%	1,134 (2.3)	545 (1.8)	251 (2.5)	183 (3.3)	155 (4.4)
Age, years	52.44 ± 11.75	51.49 ± 12.10	53.11 ± 11.15	54.50 ± 10.82	55.46 ± 10.73
Male, *n*%	37,851 (76.2)	23,068 (75.6)	7,797 (77.8)	4,299 (77.0)	2,687 (75.8)
Physic, *n*%	7,038 (14.2)	4,028 (13.2)	1,502 (15.0)	898 (16.1)	610 (17.2)
Smoke, *n*%	17,082 (34.4)	9,976 (32.7)	3,603 (35.9)	2,104 (37.7)	1,399 (39.4)
Drink, *n*%	3,054 (6.1)	1,677 (5.5)	683 (6.8)	421 (7.5)	273 (7.7)
HTN, *n*%	21,816 (43.9)	12,046 (39.5)	4,817 (48.0)	2,945 (52.7)	2,008 (56.6)
Systolic BP, mmHg	130.32 ± 19.12	128.41 ± 19.77	131.91 ± 18.91	134.07 ± 19.12	136.39 ± 20.11
Diastolic BP, mmHg	84.22 ± 10.81	83.33 ± 10.61	85.15 ± 10.81	85.84 ± 10.88	86.64 ± 11.11
DM, *n*%	4,997 (10.1)	2,331 (7.6)	1,159 (11.6)	885 (15.8)	622 (17.5)
FBG, mmol/L	5.63 ± 1.72	5.48 ± 1.62	5.73 ± 1.70	5.97 ± 1.91	6.08 ± 2.05
TC, mmol/L	4.99 ± 1.20	4.52 ± 0.674	5.3 ± 1.45	5.92 ± 1.45	6.60 ± 0.97
HDL-C, mmol/L	1.56 ± 0.50	1.56 ± 0.496	1.57 ± 0.508	1.56 ± 0.46	1.55 ± 0.45
LDL-C, mmol/L	2.62 ± 0.93	2.37 ± 0.838	2.79 ± 0.865	3.07 ± 0.85	3.48 ± 1.00
TG, mmol/L	1.70 ± 1.70	1.38 ± 1.11	1.92 ± 1.96	2.43 ± 2.50	2.66 ± 2.55
Non-HDL-C, mmol/L	3.43 ± 1.20	2.97 ± 0.704	3.73 ± 1.45	4.36 ± 1.41	5.05 ± 0.861
BMI, kg/m^2^	25.08 ± 3.38	24.72 ± 3.37	25.47 ± 3.31	25.80 ± 3.36	26.03 ± 3.24
Lipid-lowing drugs, *n*%	782 (1.6)	298 (1.0)	219 (2.2)	143 (2.6)	122 (3.4)
Antihypertensive drugs, *n*%	5,003 (10.1)	2,381 (7.8)	1,180 (11.8)	837 (15.0)	605 (17.1)
Hypoglycemic drugs, *n*%	1,511 (3.0)	674 (2.2)	389 (3.9)	283 (5.1)	165 (4.7)

ASCVD, atherosclerotic cardiovascular disease; BP, blood pressure; BMI, body mass index; DM, diabetes mellitus; FBG, fasting blood glucose; HTN, hypertension; HDL-C, high-density lipoprotein cholesterol; LDL-C, low-density lipoprotein cholesterol; non-HDL-C, non-high-density lipoprotein cholesterol; TC, total cholesterol; TG, triglyceride.

**Table 2:** Characteristics of participants by cumNon-HDL-C concentration.

Characteristics	Total	Quintiles of cumNon-HDL-C concentration (mmol/L)				
		<10.93	10.93–12.68	12.69–14.32	14.33–16.72	≥16.73
Subjects, *n*%	49,679	9,954 (20.0)	9,918 (20.0)	9,950 (20.0)	9,920 (20.0)	9,937 (20.0)
ASCVD, *n*%	1,134 (2.3)	144 (1.4)	169 (1.7)	207 (2.1)	245 (2.5)	369 (3.7)
Age, years	52.44 ± 11.75	48.87 ± 11.76	50.33 ± 11.56	51.95 ± 11.48	54.23 ± 11.19	56.81 ± 11.04
Male, *n*%	37,851 (76.2)	7,133 (71.7)	7,626 (76.9)	7,947 (79.9)	7,690 (77.5)	7,455 (75.0)
Physic, *n*%	7,038 (14.2)	1,216 (12.2)	1,268 (12.8)	1,245 (12.5)	1,548 (15.6)	1,761 (17.7)
Smoke, *n*%	17,082 (34.4)	3,362 (33.8)	3,474 (35.0)	3,501 (35.2)	3,413 (34.4)	3,332 (33.5)
Drink, *n*%	3,054 (6.1)	581 (5.8)	613 (6.2)	625 (6.3)	631 (6.4)	604 (6.1)
HTN, *n*%	21,816 (43.9)	3,314 (33.3)	3,796 (38.3)	4,411 (44.3)	4,872 (49.1)	5,423 (54.6)
Systolic BP, mmHg	130.32 ± 19.12	125.03 ± 18.14	127.79 ± 18.0	130.37 ± 18.34	132.84 ± 19.08	135.63 ± 20.14
Diastolic BP, mmHg	84.22 ± 10.81	81.75 ± 10.70	83.39 ± 10.60	84.70 ± 10.39	85.30 ± 10.77	85.97 ± 11.08
DM, *n*%	4,997 (10.1)	586 (5.9)	722 (7.3)	924 (9.3)	1,199 (12.1)	1,566 (15.8)
FBG, mmol/L	5.63 ± 1.72	5.34 ± 1.91	5.47 ± 1.35	5.61 ± 1.42	5.79 ± 1.91	5.96 ± 1.83
TC, mmol/L	4.99 ± 1.20	4.13 ± 0.69	4.58 ± 0.62	4.90 ± 0.64	5.29 ± 0.74	6.04 ± 1.81
HDL-C, mmol/L	1.56 ± 0.50	1.63 ± 0.66	1.54 ± 0.44	1.53 ± 0.43	1.55 ± 0.44	1.56 ± 0.45
LDL-C, mmol/L	2.62 ± 0.93	2.07 ± 0.63	2.42 ± 1.12	2.59 ± 0.65	2.82 ± 0.76	3.17 ± 0.97
TG, mmol/L	1.70 ± 1.70	1.24 ± 1.08	1.46 ± 1.32	1.65 ± 1.70	1.88 ± 1.65	2.28 ± 2.30
Non-HDL-C, mmol/L	3.43 ± 1.20	2.50 ± 0.85	3.04 ± 0.59	3.37 ± 0.60	3.74 ± 0.68	4.48 ± 1.75
BMI, kg/m^2^	25.08 ± 3.38	24.13 ± 3.38	24.79 ± 3.35	25.15 ± 3.29	25.58 ± 3.35	25.78 ± 3.28
Lipid-lowing drugs, *n*%	782 (1.6)	82 (0.8)	99 (1.0)	126 (1.3)	174 (1.8)	301 (3.0)
Antihypertensive drugs, *n*%	5,003 (10.1)	636 (6.4)	708 (7.1)	868 (8.7)	1,164 (11.7)	1,627 (16.4)
Hypoglycemic drugs, *n*%	1,511 (3.0)	169 (1.7)	210 (2.1)	274 (2.8)	349 (3.5)	509 (5.1)

ASCVD, atherosclerotic cardiovascular disease; BP, blood pressure; BMI, body mass index; DM, diabetes mellitus; FBG, fasting blood glucose; HTN, hypertension; HDL-C, high-density lipoprotein cholesterol; LDL-C, low-density lipoprotein cholesterol; non-HDL-C, non-high-density lipoprotein cholesterol; TC, total cholesterol; TG, triglyceride.

### The occurrence of ASCVD

**Figure 2:**
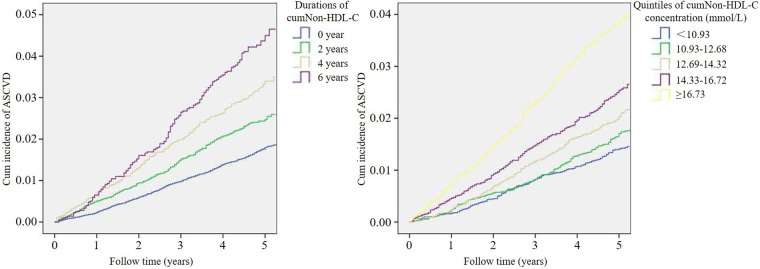
Kaplan-Meier curves of ASCVD incidence by cumulative burden of non-HDL-C.

During a mean of 4.89 years of follow-up, 1,134 participants (2.3%) had an ASCVD event, including 285 with myocardial infarction and 849 with stroke. The ASCVD incidence rate was higher with increasing cumNon-HDL-C duration from 1.8% in 0 year to 2.5%, 3.3% and 4.4% in 2, 4 and 6 years respectively. Meanwhile, the incidence rate of ASCVD was also higher with increasing quintile of cumNon-HDL-C concentration from 1.4% in the lowest quintile to 1.7%, 2.1%, 2.5% and 3.7% in the second, third, forth and fifth quintiles respectively. Likewise, Kaplan-Meier curves showed ASCVD event rates increased stepwise with increasing cumulative non-HDL-C burden (Figure 2).

### Individual cumulative non-HDL-C burden and risk of ASCVD

Multivariable Cox regression analysis revealed that compared with no exposure (duration of cumNon-HDL-C: 0 year), cumNon-HDL-C duration 2, 4 and 6 years increased ASCVD risk by 26% (HR: 1.26, 95% CI: 1.07–1.47), 56% (HR: 1.56, 95% CI: 1.31–1.86) and 91% (HR: 1.91, 95% CI: 1.59–2.31) respectively after adjusting for age, gender, cigarette smoking, alcohol consumption, physical activity, systolic BP, FBP, BMI and lipid-lowing drugs use (Table 3). The HRs (95% CIs) of cumNon-HDL-C concentration quartiles for predicting ASCVD development were 1 (ref), 1.08 (0.86–1.36), 1.20 (0.96–1.49), 1.25 (1.01–1.55) and 1.72 (1.40–2.11). When cumNon-HDL-C concentration was examined as a continuous variable, each 1-SD increment in cumNon-HDL-C concentration was associated with a 10% (HR: 1.10, 95% CI: 1.06–1.13) increased risk of ASCVD (Table 3).

**Table 3:** Relationship of cumulative non-HDL-C burden to ASCVD incidence.

	HR (95% CI) of ASCVD			
	Model 1	Model 2	Model 3	Model 4
**Durations of cumNon-HDL-C**				
0 year	1 (ref)	1 (ref)	1 (ref)	1 (ref)
2 years	1.42 (1.23–1.65)	1.37 (1.18–1.59)	1.26 (1.07–1.47)	1.21 (1.04–1.42)
4 years	1.90 (1.61–2.25)	1.75 (1.48–2.07)	1.56 (1.31–1.86)	1.47 (1.23–1.76)
6 years	2.55 (2.13–3.05)	2.28 (1.90–2.72)	1.91 (1.59–2.31)	1.76 (1.45–2.13)
*P* trend	<0.001	<0.001	<0.001	<0.001
**Quintiles of cumNon-HDL-C (mmol/L)**				
<10.93	1 (ref)	1 (ref)	1 (ref)	1 (ref)
10.93–12.68	1.20 (0.96–1.50)	1.10 (0.88–1.37)	1.08 (0.86–1.36)	1.05 (0.84–1.32)
12.69–14.32	1.49 (1.20–1.84)	1.26 (1.01–1.55)	1.20 (0.96–1.49)	1.15 (0.93–1.44)
14.33–16.72	1.81 (1.48–2.23)	1.42 (1.16–1.75)	1.25 (1.01–1.55)	1.18 (0.95–1.46)
≥16.73	2.86 (2.36–3.47)	2.03 (1.67–2.47)	1.72 (1.40–2.11)	1.56 (1.27–1.93)
*P* trend	<0.001	<0.001	<0.001	<0.001
Increase per SD	1.13 (1.10–1.15)	1.11 (1.08–1.14)	1.10 (1.06–1.13)	1.07 (1.02–1.12)
*P* trend	<0.001	<0.001	<0.001	0.007

Model 1 unadjusted. Model 2 adjusted for age, gender. Model 3 adjusted for age, gender, cigarette smoking, alcohol consumption, physical activity, systolic BP, FBP, BMI and lipid-lowing drugs use at baseline. Model 4 adjust for model 3 and non-HDL-C at baseline. ASCVD, atherosclerotic cardiovascular disease; HR, hazard ratio; non-HDL-C, non-high-density lipoprotein cholesterol; SD, standard deviation.

In order to explore whether the hazard of cumulative non-HDL-C burden to ASCVD was affected by a single non-HDL-C measurement, we further adjusted the value of non-HDL-C on 2006. The results showed that compared with no exposure, the adjusted HRs (95% CIs) for ASCVD at 2, 4, and 6 years of cumNon-HDL-C duration were 1.21 (1.04–1.42), 1.47 (1.23–1.76) and 1.76 (1.45–2.13) respectively (Table 3). The HR for the fifth versus first quintile of cumNon-HDL-C concentration was 1.56 (*P *< 0.001) for ASCVD (Table 3).

### Sensitivity analysis

To eliminate the mixed effects of antihypertensive drugs, hypoglycemic drugs and lipid-lowing drugs use on the association between cumulative non-HDL-C burden and ASCVD, participants who used these drugs were deleted respectively. The results were similar to the analysis performed on the whole participant population (Table 4). Multivariable adjusted analysis showed that among participants who did not take antihypertensive drugs, hypoglycemic drugs and lipid-lowing drugs, compared with no exposure, the adjusted HRs of cumNon-HDL-C duration 2, 4 and 6 years for ASCVD were 1.25 (1.04–1.49), 1.51 (1.23–1.86), and 2.03 (1.63–2.51) respectively (Table 4). The HRs for the fourth and fifth versus first quintile of cumNon-HDL-C concentration were 1.31 (1.02–1.67), 1.83 (1.45–2.31) for ASCVD respectively (Table 4).

**Table 4:** Relationship of cumulative non-HDL-C burden to ASCVD incidence (sensitive analyses).

	HR (95% CI) of ASCVD			
	Model 5	Model 6	Model 7	Model 8
**Durations of cumNon-HDL-C**				
0 year	1 (ref)	1 (ref)	1 (ref)	1 (ref)
2 years	1.29 (1.09–1.54)	1.25 (1.06–1.47)	1.27 (1.08–1.48)	1.25 (1.04–1.49)
4 years	1.56 (1.28–1.91)	1.53 (1.28–1.84)	1.55 (1.30–1.85)	1.51 (1.23–1.86)
6 years	2.04 (1.65–2.52)	1.99 (1.64–2.41)	1.91 (1.58–2.31)	2.03 (1.63–2.51)
*P* trend	<0.001	<0.001	<0.001	<0.001
**Quintiles of cumNon-HDL-C (mmol/L)**				
<10.93	1 (ref)	1 (ref)	1 (ref)	1 (ref)
10.93–12.68	1.18 (0.91–1.52)	1.09 (0.86–1.37)	1.10 (0.87–1.40)	1.19 (0.92–1.53)
12.69–14.32	1.24 (0.96–1.59)	1.16 (0.92–1.45)	1.20 (0.96–1.50)	1.18 (0.92–1.52)
14.33–16.72	1.35 (1.06–1.72)	1.23 (0.98–1.53)	1.25 (1.01–1.56)	1.31 (1.02–1.67)
≥16.73	1.90 (1.50–2.39)	1.70 (1.38–2.10)	1.75 (1.42–2.15)	1.83 (1.45–2.31)
*P* trend	<0.001	<0.001	<0.001	<0.001
Increase per SD	1.10 (1.06–1.14)	1.09 (1.06–1.13)	1.10 (1.06–1.14)	1.10 (1.06–1.14)
*P* trend	<0.001	<0.001	<0.001	0.007

Model 5 adjusted for model 3 and further excluded antihypertensive drugs. Model 6 adjusted for model 3 and further excluded hypoglycemic drugs. Model 7 adjusted for model 3 and further excluded lipid-lowing drugs use at baseline. Model 8 adjusted for model 3 and further excluded antihypertensive drugs, hypoglycemic drugs and lipid-lowing drugs use at baseline. ASCVD, atherosclerotic cardiovascular disease; HR, hazard ratio; non-HDL-C, non-high-density lipoprotein cholesterol; SD, standard deviation.

## Discussion

We characterized cumulative non-HDL-C burden including long-term and higher cumNon-HDL-C was significantly associated with increased rates of ASCVD development. Adjusting for use of lipid-lowing drugs and a single non-HDL-C level at baseline did not substantially attenuate these associations. These finding provide additional evidence that long-term exposure to high non-HDL-C is more likely to develop ASCVD.

In general, LDL-C, non-HDL-C, and ApoB provide similar effects on ASCVD risk (1, 8, 18). Under certain circumstances, particularly in people with high triglyceride levels, diabetes mellitus, obesity, or very low LDL-C levels, non-HDL-C evaluation is recommended for risk assessment due to the potential inaccuracy of LDL-C (11). Individuals treated with statins who achieve low LDL-C levels but have high concentrations of either non-HDL-C or ApoB remain at increased cardiovascular risk (3). REVEAL-HPS3 illustrated that after the application of anacetrapib to patients with achieved LDL-C, the reduction in the risk of myocardial infarction was consistent with the proportion of non-HDL-C decrease (19). Accumulating evidence indicates that non-HDL-C may be superior to LDL-C for prediction of cardiovascular disease risk.

The key initiating process in atherogenesis is the subendothelial retention of ApoB-containing lipoproteins (20). Atherosclerotic plaques grow over time as additional ApoB-containing lipoprotein particles are retained. The size of the total atherosclerotic plaque burden is likely to be determined by both the concentration of circulating ApoB-containing lipoproteins, and by the total duration of exposure to these lipoproteins. Therefore, a person's total atherosclerotic plaque burden is likely to be proportional to the cumulative exposure to these lipoproteins (21). Non-HDL-C offers a way to analyze the total amount of proatherogenic lipoproteins containing ApoB (1, 2). Increased non-HDL cholesterol blood concentrations early in life seem to be stable over the life course and are predictive for incident cardiovascular disease (22). We are the first to clarify the impact of cumulative non-HDL-C burden including duration and concentration of cumNonHDL-C on ASCVD. Compare with no exposure, cumNon-HDL-C duration 6 years increased ASCVD risk by up to 91% after adjusting for confounding factors. The fifth quartile of cumNon-HDL concentration increased ASCVD risk by 72% compare with the lowest quartile. In addition, to explore whether the effect of cumulative non-HDL-C burden on ASCVD was influenced by single non-HDL-C concentration, we added the adjustment of single non-HDL-C level and found that the risks were similar as before. Therefore, cumulative non-HDL-C burden was significantly associated with increased rates of ASCVD development independent of single non-HDL-C level.

This study has several limitations. First, some unmeasured confounders such as dietary fatty acids and carbohydrates (23), oils and solid fats (24) and some diseases include hepatitis (25) and nephrotic syndrome may have effects on the association of non-HDL-C and ASCVD. Second, Our study has unbalanced distributions of gender while the sex-specific effect of lipid-related biomarkers has been evaluated (26). Third, the sample was predominantly Chinese, and thus this finding may not be consistent in other racial groups.

In this Kailuan study, we provide strong support for the concept that cumulative non-HDL-C burden has both a causal and cumulative effect on the risk of ASCVD. Long-term and higher cumNon-HDL-C were all significantly associated with an increased risk of ASCVD independent of single non-HDL-C level. This also provides the rationale for encouraging a healthy lifestyle to maintain long-term low levels of non-HDL-C to slow the progression of atherosclerosis and prevent cardiovascular events. These data could be useful for physician-patient communication about primary prevention strategies.

## Data Availability

The raw data supporting the conclusions of this article will be made available by the authors, without undue reservation.
